# Effects of Caffeine and Coffee on Human Functioning

**DOI:** 10.3390/nu12010125

**Published:** 2020-01-02

**Authors:** Juan Del Coso, Juan José Salinero, Beatriz Lara

**Affiliations:** 1 Centre for Sport Studies, Rey Juan Carlos University, Fuenlabrada, 28943 Madrid, Spain; 2 Exercise Physiology Laboratory, Camilo José Cela University, 28692 Madrid, Spain; jjsaalinero@ucjc.edu (J.J.S.); blara@ucjc.edu (B.L.)

As expected, 2019 has been a prolific year in terms of new evidence regarding the effects of coffee and caffeine consumption on diverse aspects of human functioning. A search in PubMed for published studies in 2019 on the effects of caffeine or coffee on humans, following the Preferred Reporting Items for Systematic Review and Meta-Analyses (PRISMA) guidelines [1], showed a total of 202 manuscripts that contained “coffee” (n = 65, which represents 32.2% of the total) or “caffeine” (n = 137, which represents 67.8% of the total) in the title of the manuscript (Figure 1). In the group of studies that investigated the effect of coffee intake, 58 (89.2%) were related to the use of this beverage to modify one or more health outcomes, five (7.7%) were related to the use of coffee to improve human performance and two (3.1%) assessed regular intake of coffee. In the group of studies that investigated the effect of caffeine intake (in most cases measured as the sum of all the sources containing caffeine such as coffee, tea, chocolate, energy drinks, etc.), 79 (57.7%) were associated with the use of caffeine with health variables, 52 (38.0%) were associated with the use of caffeine with ergogenic purposes, six (4.4%) were associated with regular caffeine intake. Briefly, this analysis shows the elevated amount of new information published each year regarding the utility of coffee and caffeine to produce a change in human functioning while reveals that most of the indications of coffee and caffeine are associated with producing a benefit on health or with enhancing human performance.

This special edition in Nutrients has brought together a variety of investigation that imitates the pattern of published manuscripts commented above. This issue entitled “Coffee and Caffeine Consumption for Human Health” gathered 20 manuscripts; two (10.0%) were associated with coffee intake and 18 (90%) were associated with caffeine intake. In the manuscripts associated with the use of coffee, one original investigation was geared to study the perceptions of consumers regarding the health benefits that they might obtain with the regular consumption of this beverage [2]. Interestingly, 75.2% of the study sample perceived coffee as negative for their health, while the investigation determined that coffee users that seek potential health benefits of coffee are more likely to be male, young, and working. The other investigation associated with coffee intake was a systematic review and meta-analysis of prospective studies on the effect of this beverage on the risk of colorectal cancer [3]. In this study, a total of 26 investigations were analyzed while the main finding was a weak but significant protective effect of habitual coffee intake on the risk of suffering colon cancer. In addition, the regular intake of decaffeinated coffee exerted a protective effect against colorectal cancer, suggesting that part of the positive effect of coffee to reduce the risk of suffering colorectal cancer is independent of caffeine. Both investigations reflect the beliefs and patterns of our society because evidence shows that the regular intake of coffee can have a positive impact on several health outcomes [4]. Nevertheless, consumers are still cautious about drinking coffee because of the negative image of coffee-(particularly caffeinated coffee), which is not based on the latest scientific evidence [2]. More efforts should be made to translate to our society the new pieces of evidence that support the positive effect of regular coffee consumption on health, in addition to the caution that should be taken in terms of dose, interactions with other substances, and prevalence of side-effects (e.g., stimulant-like effects).

The remaining 18 studies of this issue investigated the effect of caffeine. There was a particular focus on the ergogenic effect of caffeine as 14 (77.8% of the investigations with caffeine in this special issue) investigations were related to this topic. The amount of caffeine ingested on a regular basis was associated with two (11.1%), and the remaining two (11.1%) determined the effect of caffeine on health variables. In the investigations that studied caffeine’s ergogenicity, several shared a common message because they reflect that the acute intake of caffeine (from ~1 to ~6 mg/kg of body mass) was effective to improve different aspects of physical and sport performance [5,6,7,8,9], along with enhancement in reaction times and psychological parameters [6]. In addition, several investigations responded to an Editorial [10] that fostered investigations to assess the effect of acute caffeine intake in female athletes because most of the current knowledge about the caffeine’s ergogenicity is based on investigations carried out with only-male study samples. As an answer to this call, Mielgo-Ayuso et al. [11] presented an analysis, based on a systematic review, indicating that acute caffeine intake exhibited a similar ergogenic benefit for aerobic performance in men and women athletes. However, the ergogenic effect of caffeine was inferior in women than in men in strength- and power-based tests, even when the same dose of caffeine was being administered. This significant, although low in magnitude, effect of caffeine to increase muscle power and force in women was confirmed by Romero-Moraleda [12], but these authors suggested that caffeine’s ergogenicity was similar across the menstrual cycle (by investigating placebo-caffeine comparisons in the early follicular, late follicular and mid-luteal phases). All these investigations have contributed to explaining the effect of caffeine on human performance, which is present in several exercise situations and with several dosages, although further investigations should be carried out to explain the individual differences in the magnitude of the ergogenic effect of caffeine [13].

The clear evidence provided by this special issue confirming the ergogenic effect of caffeine might be behind the slight increase in the use of caffeine in sports since its removal from the list of banned substances in 2004 [14]. By analyzing the concentration of caffeine in post-competition urine samples, it has been found that about three out of four athletes consume caffeine or caffeine-containing products to increase performance [14]. Interestingly, the investigation by Shabir et al. [15], who used a double-dissociation experimental design where caffeine and a placebo were administered in situations in which participants were informed or misinformed of the substance that they had ingested, determined that part of the ergogenic effect of caffeine on human performance is explained by the psychological impact of the expectancy of ergogenicity that caffeine produces in athletes. Thus, believing to have ingested caffeine, or feeling the stimulation that it produces, might be an important part of the actual ergogenic effect of caffeine [16]. In this regard, caffeine ergogenicity can be obtained by the synergistic action of the pharmacological effect of this substance on the central nervous system [8] and in other peripheral tissues [17], together with the psychological effect of this potent stimulant [15].

Nevertheless, habituation to caffeine through the regular intake of this substance might be an important modifier for the obtaining of caffeine ergogenicity. The ingestion of 6 mg/kg of caffeine did not improve the time employed to complete an 800 m competition in athletes habituated to caffeine while it negatively affected sleep quality [18]. Similarly, low-to-moderate doses of caffeine (from 3 to 9 mg/kg), were found to be ergogenic in other situations with individuals who do not consume caffeine or are low caffeine consumers [19,20] and seemed ineffective in increasing muscle performance in athletes habituated to caffeine intake [21]. These two investigations [18,21] indicate that the use of moderate doses of caffeine might not be ergogenic in individuals habituated to caffeine, likely due to the progressive tolerance to the ergogenic effect of this substance when it is ingested chronically [22]. For athletes habituated to caffeine, the use of high doses (up to 11 mg/kg) might exert a positive effect on maximal strength values, but may negatively affect muscle endurance while increasing the prevalence of caffeine-induced drawbacks [23]. All this information taken together suggests that athletes who are consuming caffeine in a habitual manner should refrain from caffeine intake for several days to remove/reduce tolerance to the ergogenic effect of this substance. For athletes habituated to caffeine who seek caffeine’s ergogenicity, the dishabituation to caffeine is recommended instead of using doses of caffeine higher than the daily habitual intake.

Other contributions to science published in this issue suggest the possibility of using the measurement of urinary caffeine metabolites as a routine clinical examination for evaluating drug metabolic phenotypes [24], the harmful effects of the administration of high doses of caffeine on the adrenal glands of immature rats [25], and the safety of a mean caffeine intake <200 mg/day to avoid any effect on neonatal weight, length, or head, and chest circumference [26].

The diversity of the articles published in this special issue highlights the extent of the effects of coffee and caffeine on human functioning while it underpins the positive nature of most of these effects. More work is necessary to completely understand the complex mechanisms behind each effect of caffeine on body tissues, although this issue has greatly contributed to unveil how coffee and caffeine might be used to improve human functioning.

## Figures and Tables

**Figure 1:**
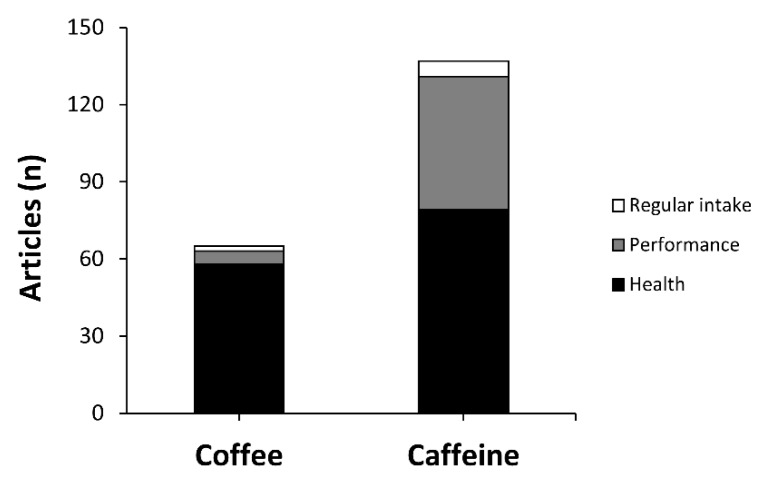
Number of articles published in 2019 that investigated the effects of coffee or caffeine on humans.
